# P7C3 suppresses astrocytic senescence to protect dopaminergic neurons: Implication in the mouse model of Parkinson’s disease

**DOI:** 10.1111/cns.14819

**Published:** 2024-07-26

**Authors:** Yajing Chen, Zengyan Zhu, Yinghui Yan, Hongyang Sun, Guanghui Wang, Xiaohuan Du, Fang Li, Shuwei Yuan, Wenjing Wang, Mei Wang, Chao Gu

**Affiliations:** ^1^ Department of Pharmacy Children's Hospital of Soochow University Suzhou China; ^2^ Laboratory of Molecular Neuropathology, Jiangsu Key Laboratory of Translational Research and Therapy for Neuropsychiatric Disorders, Department of Pharmacology, College of Pharmaceutical Sciences Soochow University Suzhou Jiangsu China

**Keywords:** astrocyte, Parkinson's disease, PC73, senescence, Sirt3

## Abstract

**Aims:**

Astrocytic senescence is inextricably linked to aging and neurodegenerative disorders, including Parkinson's disease (PD). P7C3 is a small, neuroprotective aminopropyl carbazole compound that exhibits anti‐inflammatory properties. However, the effects of P7C3 on astrocytic senescence in PD remain to be elucidated.

**Methods:**

An in vitro, long culture‐induced, replicative senescence cell model and a 1‐methyl‐4‐phenylpyridinium (MPP^+^)/rotenone‐induced premature senescence cell model were used to investigate the effects of P7C3 on astrocytic senescence. An in vivo, 1‐methyl‐4‐phenyl‐1,2,3,6‐tetrahydropyridine (MPTP)‐induced mouse PD model was used to study the role of P7C3 in astrocytic senescence. Immunoblotting, real‐time quantitative RT‐PCR (qPCR), immunofluorescence, subcellular fractionation assays, and immunohistochemistry were utilized to confirm the effects of P7C3 on astrocytic senescence and elucidate its underlying mechanisms.

**Results:**

This study determined that P7C3 suppressed the senescence‐associated secretory phenotype (SASP) in both cell models, as demonstrated by the reduction in the critical senescence marker p16 and proinflammatory factors (*IL‐6*, *IL‐1β*, *CXCL10*, and *MMP9*) and increased laminB1 levels, implying that P7C3 inhibited replicative astrocytic senescence and MPP^+^/rotenone‐induced premature astrocytic senescence, Most importantly, we demonstrated that P7C3 prevented the death of dopamine (DA) neurons and reduced the behavioral deficits in the MPTP‐induced mouse model of PD, which is accompanied by a decrease in senescent astrocytes in the substantia nigra compacta (SNc). Mechanistically, P7C3 promoted Nrf2/Sirt3‐mediated mitophagy and reduced mitochondrial reactive oxygen species (mitoROS) generation, which contributed to the suppression of astrocytic senescence. Furthermore, *Sirt3* deficiency obviously abolished the inhibitory effects of P7C3 on astrocytic senescence.

**Conclusion:**

This study revealed that P7C3 inhibited astrocytic senescence via increased Nrf2/Sirt3‐mediated mitophagy and suppression of mitoROS, which further protected against DA neuronal loss. These observations provide a prospective theoretical basis for P7C3 in the treatment of age‐associated neurodegenerative diseases, such as PD.

## Graphical abstract

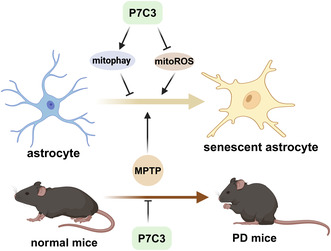

## 1 BACKGROUND

Parkinson's disease (PD), which is the second most prevalent neurodegenerative disease, is characterized by the preferential loss of dopaminergic (DA) neurons in the substantia nigra compacta (SNc).^1^ Although the pathological causes of PD are not clearly understood, a growing body of evidence suggests that abnormal astrocytes may contribute to DA neuronal death during PD.^2^, ^3^ Astrocytes are the predominant glial cells in the central nervous system (CNS), and they have critical roles in the maintenance of normal brain function, especially in neuronal homeostasis and metabolism.^4^ Thus, it is likely that astrocytic dysfunction could be involved in neuronal death.

Cellular senescence is a critical feature of aging and has been recognized as the primary component of age‐associated neurodegenerative disorders.^5^ Aging is a predominant risk factor for the development of PD.^6^ It has been reported that the occurrence rate of PD increases with advancing age.^7^ Most astrocytes become senescent during aging.^8^, ^9^ Therefore, it is likely that astrocytic senescence participates in the initiation and progression of PD. Senescent astrocytes exhibit increased production and secretion of senescence‐associated secretory phenotype (SASP) factors, including chemokines and proinflammatory cytokines, leading to the death of surrounding neurons through inflammatory damage.^10^ Moreover, senescent astrocytes lose their neuroprotective effects resulting in numerous detrimental effects for neurons, including impaired synaptic plasticity and induced glutamate excitotoxicity.^10^ Therefore, targeting senescent astrocytes might be a novel approach to treating age‐related neurodegenerative diseases, such as PD.^11^, ^12^

P7C3, a small aminopropyl carbazole compound, was initially reported to have powerful neuroprotective capabilities in newborn hippocampal neurons.^13^ P7C3 also possesses robust neuroprotective effects in specific nerve‐related diseases, including amyotrophic lateral sclerosis,^14^ retinal degeneration,^15^ traumatic brain injury,^16^, ^17^ ischemic stroke,^18^, ^19^ depressive disorder,^20^ and peripheral nerve degeneration.^21^ Interestingly, P7C3 has remarkable neuroprotective and anti‐inflammatory effects in several neurodegenerative diseases, including Alzheimer's disease (AD) and PD.^22^, ^23^ In the MPTP mouse model or the 6‐hydroxydopamine (6‐OHDA) rat model of PD, P7C3 dramatically decreased tyrosine hydroxylase (TH) neuronal loss in the SNc and striatum.^24^, ^25^ In addition, inhibition of LPS‐induced microglial activation was found in our previous study, indicating a role in neuroinflammation.^26^ However, it remains to be determined whether P7C3 is involved in regulating astrocytic senescence.

This study will shed more light on the protective role of P7C3 in PD. Our results indicated that P7C3 significantly suppressed replicative astrocytic senescence and neurotoxin‐induced premature astrocytic senescence in vitro and in vivo.

## 2 MATERIALS AND METHODS

### 2.1 Animal experiments

Six‐ to eight‐week‐old, 25–30 g C57BL/six mice were purchased from SLACCAL Lab Animal Ltd. (Shanghai, China). The mice were raised with 50%–60% relative humidity, a temperature of 20–26°C, and a 12:12 h light/dark cycle. The animals were provided water and food ad libitum. For P7C3 and MPTP treatment in vivo, the mice were randomly divided into four groups: (1) vehicle + saline, (2) P7C3 + saline, (3) vehicle + MPTP, and (4) P7C3 + MPTP. The vehicle was composed of 3% DMSO/10% cremophor EL/87.5% D5W (5% dextrose in water, pH 7.2). The mice in groups (2) and (4) were treated twice daily with 20 mg/kg/d P7C3 for 21 consecutive days via intraperitoneal injection. Mice in the other two groups were injected with an equal volume of vehicle. Then, mice in groups (3) and (4) were treated with MPTP (30 mg/kg/d) once daily for 7 days, The mice in groups (1) and (2) were treated with the same volume of saline. The mice were euthanized 24 h after treatment. The midbrains were removed and homogenized in TRIZOL buffer, and total RNA was isolated using an RNA extraction kit (Takara, Otsu, Shiga, Japan) according to the manufacturer's recommendations. All animal experiments were performed according to the Regulations of the Experimental Animal Administration issued by the Animal Committee of Soochow University.

### 2.2 Behavioral tests

Behavioral experiments, including the rotarod and pole climbing tests, were performed 1 week after the last MPTP treatment. The behavioral apparatuses were purchased from SANS Biological Technology (Nanjing, China). The training and testing methods were carried out. All behavioral tests were evaluated in the same animal facilities.

#### 2.2.1 Rotarod test

For training, each mouse was placed on the rotarod and trained until it could run on the rotarod for more than 2 min at a speed of 4 revolutions per minute (r/min). For testing, we accelerated the rotarod speed from 4 r/min to 40 r/min over 5 min. Each mouse ran freely on the rotarod until it fell, and the time to fall was recorded. Each mouse performed the rotarod test three times, and the average times were calculated.

#### 2.2.2 Pole test

A wooden pole with a height of 50 cm, a diameter of 1 cm, and a rough surface was placed in the cage. On the training day, each mouse was placed on the top of the pole and allowed to climb down from the top of the pole into its cage. This was repeated three times. On the testing day, each mouse was placed head‐down on a perch, and the time it took for the mouse to fall into its cage was recorded. The experiment was repeated three times for each mouse, and the average times were utilized in the analysis.

### 2.3 Primary astrocyte culture and treatment

Primary cultured astrocytes were obtained as described below. Briefly, brain tissue was removed from neonatal 3‐day‐old C57BL/six mice, and the blood vessels and meninges were removed. A mixed glial cell suspension was obtained after digestion and filtration. The isolated cells were inoculated into 75cm^2^ culture flasks coated with poly‐D‐lysine and cultured with DMEM/F12 (Gibco, Grand Island, NY, USA) supplemented with 10% heat‐inactivated fetal bovine serum (FBS). After 2 weeks of culture, the mixed glia was gently shaken for 2 h at 200 r/min, and the medium containing microglia was discarded. The attached astrocytes remained on the bottom of the flask and were cultured with fresh DMEM/F12. Astrocytes were pretreated with P7C3 (10 μM, MCE, NJ, USA) for 2 h before MPP^+^ (500 μM, Sigma, St. Louis, MO, USA) or rotenone (1 μM, Sigma, St. Louis, MO, USA) exposure for 24 h. Primary cultured astrocytes were passaged every four to 5 days and cultured for 40 days in vitro (DIV) to obtain replicative senescent astrocytes.^27^ Senescent astrocytes were treated with P7C3 (0.1, 1, 10 μM) for 5 days. Astrocytes (10 DIV) in the control groups also were treated with P7C3 (0.1, 1, 10 μM) for 5 days.

### 2.4 β‐Galactosidase staining

Primary astrocytes from the different treatments were washed three times with phosphate‐buffered saline (PBS) and placed in fixative at room temperature for 15 min. Senescent astrocytes were assessed with a β‐galactosidase‐based Senescence Cells Staining Kit (Beyotime Biotechnology, Shanghai, China) according to the manufacturer's instructions. Senescent astrocytes stained blue and were observed with a light microscope at PH 6. We counted the number of positively stained astrocytes and the total number of cells. The percentage of positive‐stained astrocytes compared to the total cell number was derived from three separate cultures.

### 2.5 RNA interference

RNAiMAX (Invitrogen, Waltham, MA) was utilized to transfect primary astrocytes with RNA oligonucleotides. Senescent astrocytes were incubated in a mixture of RNAiMAX, RNA oligonucleotides, and Opti‐MEM for 15 min at room temperature. Twenty‐four hours later, the medium was replaced with fresh medium. After an additional 24 h, cells were treated with P7C3. Oligonucleotides targeting mouse *Sirt3* (Si‐Sirt3, sc‐61,555) were purchased from Santa Cruz Biotechnology (Santa Cruz, CA, USA).

### 2.6 RNA extraction, reverse transcription‐PCR, and real‐time quantitative RT‐PCR

After the primary astrocyte treatments, total RNA was obtained using TRIzol reagent (Invitrogen, Carlsbad, CA, USA). We prepared cDNA using PrimeScript RT Master Mix (Takara, Otsu, Shiga, Japan). Real‐time quantitative PCR was performed using the SYBR Green PCR Master Mix (Applied Biosystems, Warrington, Cheshire, UK). The product measurements were performed using the Applied Biosystems 7500 Real‐Time PCR System (Applied Biosystems, Warrington, Cheshire, UK). The PCR primer sequences were as follows: mouse *IL‐6*: 5′‐GCTATGAAGTTCCTCTCTGC‐3′ and 5′‐CTAGGTTTGCCGAGTAGATC‐3′, mouse *IL‐1β*: 5′‐TGGCAACTGTTCCTG‐3′ and 5′‐GGAAGCAGCCCTTCATCTTT‐3′, mouse *MMP‐9*: 5′‐CGACTTTTGTGGTCTTCCCC‐3′ and 5′‐AGCGGTACAAGTATGCCTCTG‐3′, mouse CXCL10: 5′‐CCAAGTGCTGCCGTCATTTTC‐3′ and 5′‐GGCTCGCAGGGATGATTTCAA‐3′, mouse *β‐actin*: 5′‐ GACCTGACTGACTACCTC‐3′ and 5′‐GACAGCGAGGCCAGGATG‐3′.

### 2.7 Immunoblot analysis and antibody information

Phosphatase and protease inhibitors (Roche, Mannheim, Germany) were dissolved in 1 × SDS lysis buffer (150 mmol/L NaCl, 25 mmol/L Tris–HCl, pH 7.6, 1% deoxycholic acid sodium, and 1% NP‐40) to lyse primary astrocytes or isolated tissues. Approximately 20 μg aliquots of the cell lysates were separated by SDS‐PAGE and transferred to PVDF membranes (Millipore, Billerica, MA, USA). Western blot analysis was performed using the following primary antibodies: anti‐p16^Ink4a^ (Cat No. 10883‐1‐AP), anti‐LaminB1 (Cat No. 12987‐1‐AP), anti‐LC3 (Cat No. 14600‐1‐AP), anti‐p62 (Cat No. 18420‐1‐AP), anti‐NRF2 (Cat No. 16396‐1‐AP), anti‐KEAP1 (Cat No. 10503‐2‐AP), anti‐Histone 2B (Cat No. 15857‐1‐AP), anti‐α‐tubulin (Cat No. 11224‐1‐AP), anti‐HO‐1 (Cat No. 10701‐1‐AP), anti‐MAO‐B (Cat No. 12602‐1‐AP) (Proteintech, Chicago, USA); anti‐SIRT3 (Cat No. 5490S) (Cell Signaling Technology, Danvers, MA, USA); anti‐β‐actin (Cat No. ab8226), anti‐Tom20 (Cat No. ab186735), anti‐COXIV (Cat No. ab16056) (Abcam, Cambridge, UK); anti‐TH (Cat No. AB9072) (Millipore, Billerica, MA, USA). Secondary antibodies, sheep anti‐rabbit or anti‐mouse IgG‐HRP, were purchased from Thermo Fisher Scientific (Waltham, MA, USA). Proteins were visualized using an ECL detection kit (Thermo Fisher Scientific, Waltham, MA).

### 2.8 Subcellular fractionation assay

Fraction assays were performed. Briefly, after treating with P7C3, a separation medium containing 3 mM CaCl_2_, 2 mM MgAc, 320 mM sucrose, 0.1 mM EDTA, 1 mM DTT, 0.5 mM phenylmethylsulfonyl fluoride (PMSF), and 0.5% NP‐40 buffer was used to lyse the senescent astrocytes. The lysate was held on ice for 20 min. After centrifugation at 600 g for 15 min at 4°C, the supernatant was recovered as the cytoplasmic fraction. The resulting pellets were washed twice with the fractionation buffer without NP‐40 and lysed using nuclear lysis buffer that contained 280 mM KCl, 0.2 mM EDTA, 1 mM DTT, 0.5 mM PMSF, 20 mM Hepes (pH 7.9), 25% glycerol, 1.5 mM MgCl_2_, and 0.3% NP‐40 to yield the nuclear fraction.

### 2.9 Mitochondrial ROS measurement and mitophagy detection

The mitochondrial reactive oxygen species (ROS) was measured by MitoSox according to the manufacturer's instructions. Briefly, astrocytes were stained with MitoSOX (2.5 μM, Invitrogen, USA), washed at 37°C for 15 min, then washed twice with PBS. Cells were observed using an IX71 inverted microscope system (Olympus, Tokyo, Japan) or measured by flow cytometry (Cytek Aurora 3 Laser 16 V‐14B‐8R). The mitophagy level in different groups were measured using Mitophagy Detection Kit (DOJINDO, Japan) according to the manufacturer's instructions.

### 2.10 Immunofluorescence staining

After primary astrocytes underwent the different treatments, cells were fixed with 4% paraformaldehyde in PBS for 5 min, then permeabilized with 0.25% Triton X‐100 in PBS for 5 min. After washing three times with PBS, the cells were blocked with 0.5% fetal bovine serum and incubated with anti‐LaminB1 (Proteintech, Chicago, USA) for 6 h at room temperature, followed by incubation with rhodamine‐conjugated (red) secondary antibody.

### 2.11 Immunohistochemistry and imaging

After completion of the in vivo treatments, the mice were anesthetized by intraperitoneal injection of 40 mg/mL pentobarbital sodium, perfused with 20 mL of PBS via the vena cava, followed by 20 mL of 0.1 M PBS (pH 7.4) containing 4% PFA. The brains were removed, fixed in the same fixative for 3 days at 4°C, then immersed in 30% sucrose for 3 days at 4°C. The brains were cut into 20 μm sections using a cryostat (Leica CM1950, Leica Biosystems, Nussloch, Germany). Immunocytochemical staining was performed with TH primary antibody (Santa Cruz Biotechnology, Santa Cruz, CA, USA) overnight at 4°C. Then the sections were incubated with Alexa Fluor secondary antibody for 2 h at room temperature. Finally, the sections were counterstained with DAPI for 10 min to label the nuclei. The sections were photographed using an IX71 inverted microscope system (Olympus, Tokyo, Japan). Three sections from each of three animals were utilized for statistical analysis. The fluorescence intensities were analyzed using ImageJ software (National Institutes of Health, Bethesda, MD, USA). Sholl analysis was conducted on astrocytic morphology following established protocols. Immunostained images of SNpc slices with GFAP antibody were captured and analyzed. The Sholl analysis plugin in ImageJ was used to get the astrocytic morphology in black and white and measure the Soma diameter of astrocytes.

### 2.12 Immunoprecipitation

Immunoprecipitation (IP) was performed by lysing cells in cell lysis buffer at 4°C, followed by centrifugation at 12,000 × g for 15 min. The resulting supernatants were then subjected to IP using appropriate antibodies coupled to protein G Sepharose beads (Roche, Basel, Basel‐City, Switzerland). The immunoprecipitates were washed with cell lysis buffer and analyzed by immunoblot. The input sample represented 10% of the original supernatant. To investigate the interaction between KEAP1 and NRF2, supernatants from astrocytes transfected with FLAG‐KEAP1 were used for IP with an anti‐FLAG antibody.

### 2.13 Statistical analysis

Results were quantified, statistically analyzed and graphed using Photoshop 7.0 (Adobe, San Jose, CA, USA) and GraphPad Prism 8.00 (GraphPad Software, Release X, La Jolla, CA, USA). All data distributions were tested for normality using Shapiro–Wilk test in GraphPad Prism 8.00. All data are presented as mean ± standard error of the mean (SEM). Statistical significance was analyzed by one‐way analysis of variance (ANOVA) with Dunnett's multiple comparison test or Tukey's multiple comparison test. Student's *t*‐tests were used to compare only two groups. *p* values below 0.05 were considered statistically significant.

## 3 RESULTS

### 3.1 P7C3 attenuated astrocytic senescence and DA neuronal loss in a MPTP‐induced PD mouse model

**FIGURE 1:**
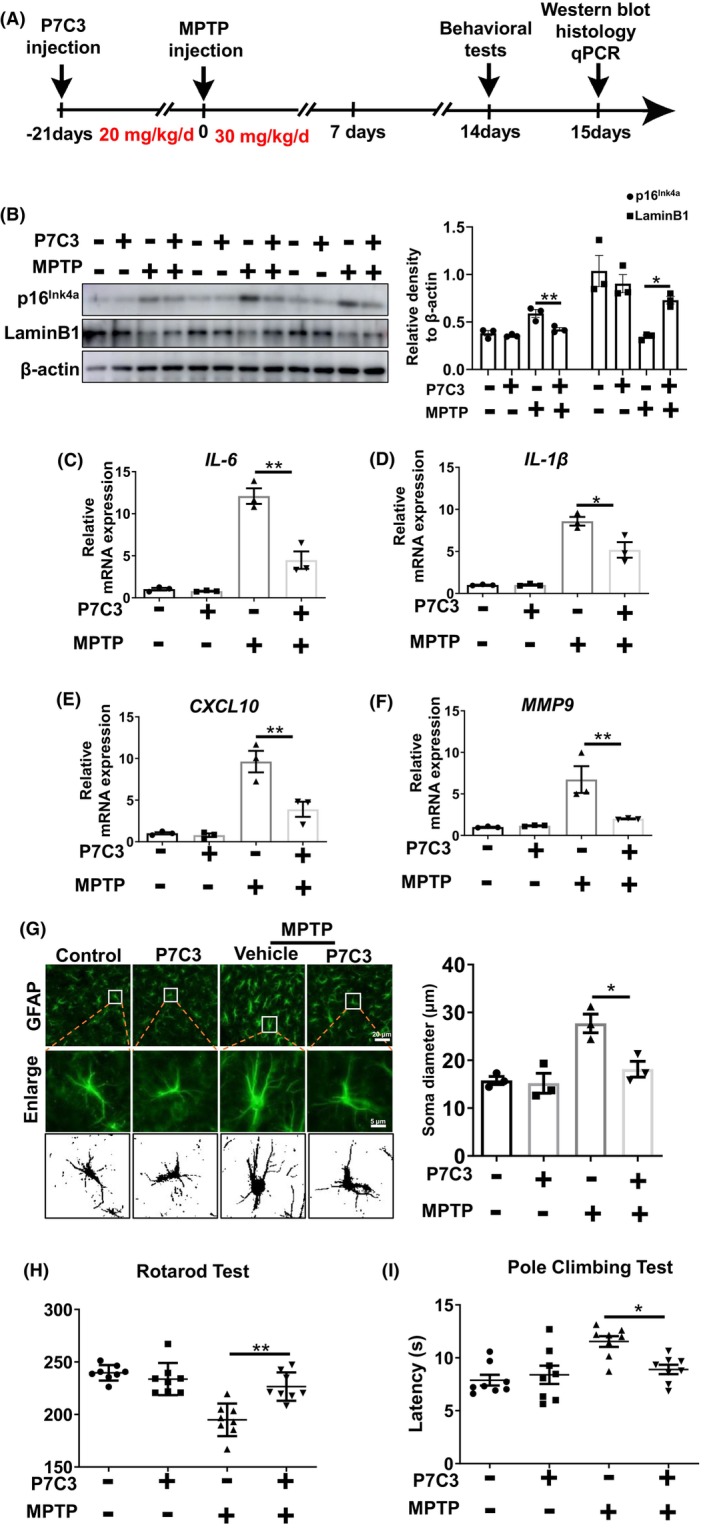
P7C3 Attenuates astrocytic senescence and DA neuronal loss in MPTP‐induced PD mouse model. (A) A schematic diagram showed the animal experimental procedure. (B) Mice SNc was isolated to collect protein for measuring protein levels of p16Ink4a, LaminB1 and β‐actin were detected by immunoblotting. The intensity quantification of p16^Ink4a^ and LaminB1 relative to β‐actin is shown in the right panel. *p < 0.05 and **p < 0.01 one‐way ANOVA followed by Dunnett's multiple‐comparisons test. (C–F) Mice SNc was isolated to collect total RNA for measuring mRNA levels of *IL‐6*, *IL‐1β*, *CXCL10*, and *MMP9* using qRT‐PCR assays. Statistical analysis was performed using one‐way ANOVA followed by Dunnett's multiple‐comparisons test, and significance was determined as *p < 0.05 and **p < 0.01 compared to the group treated with MPTP alone. (G) Immunohistochemical staining was performed using anti‐GFAP antibodies. The Soma diameter of astrocytes in different groups was quantified in the right panel. The values are presented as the mean ± SEM. *p < 0.05, one‐way ANOVA followed by Dunnett's multiple‐comparisons test. *n* = 3. (H) The delay time for the accelerated rotating beam case in the rotating rod test. Values are expressed as mean ± SEM. **p < 0.01, using Dunnett's multiple comparison test after one‐way ANOVA. (I) Climbing time on pole test. The values are presented as the mean ± SEM. *p < 0.05, one‐way ANOVA followed by Dunnett's multiple‐comparisons test.

Increasing evidence suggests that senescent astrocytes play critical roles in the pathogenesis of PD.^11^, ^12^ We established a mouse model of PD by intraperitoneal injection of MPTP once a day for 7 days. Before the MPTP injections, 21 days of intraperitoneal injections of P7C3 were carried out (Figure 1A). In the SNc of the MPTP‐induced PD mouse model, increased protein expression of the senescence‐associated marker p16^Ink4a^, and decreased LaminB1 expression were detected (Figure 1B). Moreover, the SASP factors, including *IL*‐*6*, *IL*‐*1β*, *CXCL10*, and *MMP9* increased in SNc after MPTP treatment (Figure 1C–F). The astrocytes change into a more enlarged morphology, which is a classic characteristic of senescent cells with MPTP treatment (Figure 1G). We considered whether P7C3 is capable of alleviating astrocytic senescence and further attenuating DA neuronal loss. In this study, P7C3 remarkably inhibited p16^Ink4a^ expression, recovered LaminB1 expression, decreased expression of the SASP factors (*IL‐6*, *IL‐1β*, *CXCL10*, and *MMP9*), and reversed the enlarged astrocyte morphology (Figure 1B–G). Senescent astrocytes are strikingly lethal to DA neurons. Immunohistochemistry results revealed severe TH^+^ (tyrosine hydroxylase) DA neuronal loss in the SNc of the mouse MPTP‐induced PD model. However, the TH^+^ DA neuronal loss in the SNc was significantly alleviated in the P7C3‐pretreated groups (Figure S1A,B). Moreover, in 20‐month‐old elderly mice, P7C3 could also significantly inhibit the p16^Ink4a^ expression in the SNc area (Figure S1C). Notably, we evaluated fine motor coordination using several behavioral tests, including the rotarod and pole climbing tests. Pretreatment with P7C3 shortened the rod test time and increased the rotatory rod latency, suggesting that motor deficits in MPTP‐treated mice were alleviated (Figure 1H,I). These data indicated that P7C3 rescued MPTP‐induced PD‐like pathology in mice. Thus, P7C3 reduced the accumulation of senescent astrocytes in the SNc of the MPTP‐induced PD mouse model.

### 3.2 P7C3 inhibited long‐term, culture‐induced replicative astrocytic senescence

**FIGURE 2:**
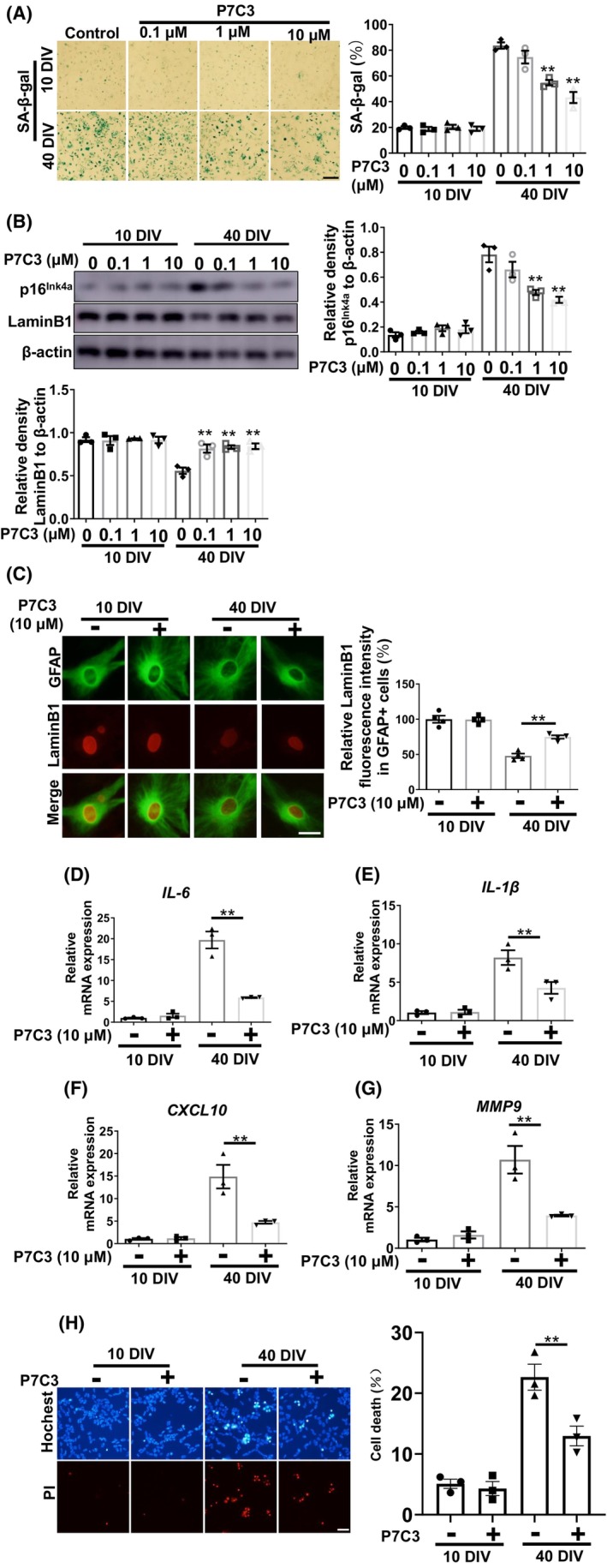
P7C3 Inhibited long‐term, culture‐induced replicative astrocytic senescence. (A) Astrocytes were cultured in vitro for 10 or 40 days and then treated with P7C3 (0.1, 1, 10 μM) for 5 days. The SA‐β‐gal was stained and the percentage of SA‐β‐gal^+^ cells was shown in the right panel. The values are presented as the mean ± SEM. **p < 0.01 compared to the 40 DIV group without P7C3 treatment, determined using one‐way ANOVA followed by Dunnett's multiple‐comparisons test. Scale bar, 50 μm (B) Astrocytes were treated as described in (A). The protein levels of p16^Ink4a^, LaminB1 and β‐actin were detected by immunoblotting. The intensity quantification of p16^Ink4a^ and LaminB1 relative to β‐actin is shown in the right panel. **p < 0.01 one‐way ANOVA followed by Dunnett's multiple‐comparisons test. (C) Astrocytes were cultured in vitro for 10 or 40 days and then treated with P7C3 (10 μM) for 5 days. Immunofluorescence staining was performed with anti‐GFAP and anti‐LaminB1, relative LaminB1 fluorescence intensity is shown the right panel. **p < 0.01 one‐way ANOVA followed by Dunnett's multiple‐comparisons test. Scale bar, 5 μm (D–G) Astrocytes were treated as described in (C). The mRNA levels of *IL‐6*, *IL‐1β*, *CXCL10M*, and *MMP9* were measured using qRT‐PCR assays. **p < 0.01 one‐way ANOVA followed by Dunnett's multiple‐comparisons test. (H) Astrocytes were treated as described in (C), and then the conditioned media from different groups were collected to culture the SHSY5Ycells for 24 h. After treatment, the SHSY5Y cells were subjected to Hoechst or PI staining to detect cell death. Scale bar, 20 μm. **p < 0.01 one‐way ANOVA followed by Dunnett's multiple‐comparisons test.

We evaluated the role of P7C3 on replicative senescence in vitro. Compared with nonsenescent astrocytes cultured for 10 days in vitro, primary astrocytes cultured for 40 days in vitro expressed characteristics of senescent astrocytes, including increased β‐galactosidase expression (Figure 2A), increased expression of the senescence‐associated marker p16^Ink4a^, and decreased LaminB1 expression (Figure 2B,C). Moreover, elevated production of typical SASP proinflammatory factors (*IL‐6*, *IL‐1β*, *CXCL10*, and *MMP9*) was detected in the long‐term cultured astrocytes. Interestingly, pretreatment with P7C3 inhibited enhanced expression of β‐galactosidase, p16^Ink4a^, and reversed the decrease in LaminB1 in a concentration gradient‐dependent manner (Figure 2A–C). More importantly, P7C3 also inhibited elevated expression of typical SASP proinflammatory factors (*IL‐6*, *IL‐1β*, *CXCL10*, and *MMP9*) in the long‐term, culture‐induced senescent astrocytes (Figure 2D–G). We speculated that P7C3 could inhibit the neurotoxicity caused by senescent astrocytes. We performed a conditioned medium (CM) assay. We prepared the CM from senescent astrocytes with or without P7C3 treatment, then were collected to culture SHSY5Y cells, a DA cell line. Thereafter, SHSY5Y cells were subjected to Hochest and propidium iodide (PI) stainings. The neurotoxicity of CM from senescent astrocytes was obviously increased, while the number of survival cells was markedly upregulated when cells were cultured with CM from senescent astrocytes that were treated with P7C3 (Figure 2H). Thus, these results suggested that P7C3 inhibited the replicative senescence of astrocytes in vitro.

### 3.3 P7C3 suppressed neurotoxin‐induced astrocytic senescence

**FIGURE 3:**
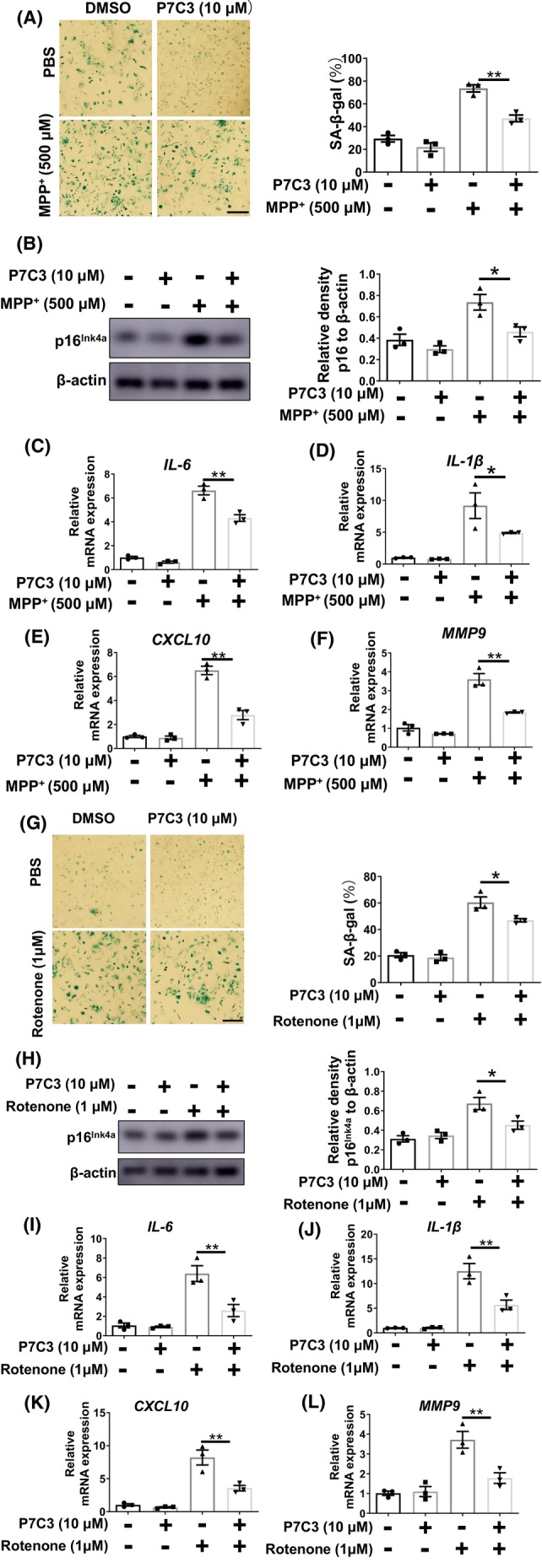
P7C3 Suppressed neurotoxin‐induced astrocytic senescence. (A) Astrocytes were pretreated with P7C3 for (10 μM) for 2 h, then were exposed to MPP^+^ (500 μM) for 24 h. The SA‐β‐gal was stained and the percentage of SA‐β‐gal^+^ cells was shown in the right panel. **p < 0.01 one‐way ANOVA followed by Dunnett's multiple‐comparisons test. Scale bar, 50 μm. (B) Astrocytes were treated as described in (A). The protein levels of p16^Ink4a^ and β‐actin and were measured using immunoblot analysis. The intensity quantification of p16^Ink4a^ relative to β‐actin is shown in the right panel. The values are presented as the mean ± SEM. *p < 0.05, one‐way ANOVA followed by Dunnett's multiple‐comparisons test. (C–F) Astrocytes were treated as described in (A). The mRNA levels of *IL‐6*, *IL‐1β*, *CXCL10*, and *MMP9* were measured using qRT‐PCR assays. **p < 0.01 one‐way ANOVA followed by Dunnett's multiple‐comparisons test. (G) Astrocytes were pretreated with P7C3 for (10 μM) for 2 h, then were exposed to Rotenone (1 μM) for 24 h. The SA‐β‐gal was stained and the percentage of SA‐β‐gal^+^ cells was shown in the right panel. Scale bar, 50 μm. (H) Astrocytes were treated as described in (G). The protein levels of p16^Ink4a^ and β‐actin and actin were measured using immunoblot analysis. The intensity quantification of p16^Ink4a^ relative to β‐actin is shown in the right panel. The values are presented as the mean ± SEM. *p < 0.05, one‐way ANOVA followed by Dunnett's multiple‐comparisons test. (I–L) Astrocytes were treated as described in (G). The mRNA levels of *IL‐6*, *IL‐1β*, *CXCL10*, and *MMP9* were measured using qRT‐PCR assays. **p < 0.01 one‐way ANOVA followed by Dunnett's multiple‐comparisons test.

PD‐associated neurotoxins, including MPP^+^ and rotenone, can trigger glial cell senescence, therefore, we evaluated the potential of P7C3 to inhibit MPP^+^/rotenone‐induced astrocytic senescence.^27^, ^28^ Cultured astrocytes exposed to MPP^+^ acquire characteristics of senescent cells, including elevated levels of β‐galactosidase (Figure 3A) and p16^Ink4a^ (Figure 3B; Figure S2A) and increased production of proinflammatory compounds, such as *IL‐6*, *IL‐1β*, *CXCL10* and *MMP9* (Figure 3C–F), which are prominent components of the SASP. Remarkably, P7C3 suppressed the increase in p16^Ink4a^ and SASP production (Figure 3A–F). We also found that P7C3 did not influence Monoamine Oxidase B (MAO‐B) expression (Figure S2B). Rotenone is a classic neurotoxin that causes PD pathogenesis and mediates senescence. We determined that P7C3 treatment reduced rotenone‐induced astrocytic senescence. Consistent with MPP^+^‐induced astrocytic senescence, P7C3 also significantly inhibited rotenone‐induced enhancement of β‐galactosidase and p16^Ink4a^ expression and the typical SASP factors (Figure 3G–L).

### 3.4 Enhanced mitophagy and reduced mitoROS are involved in P7C3‐mediated astrocytic senescence inhibition

**FIGURE 4:**
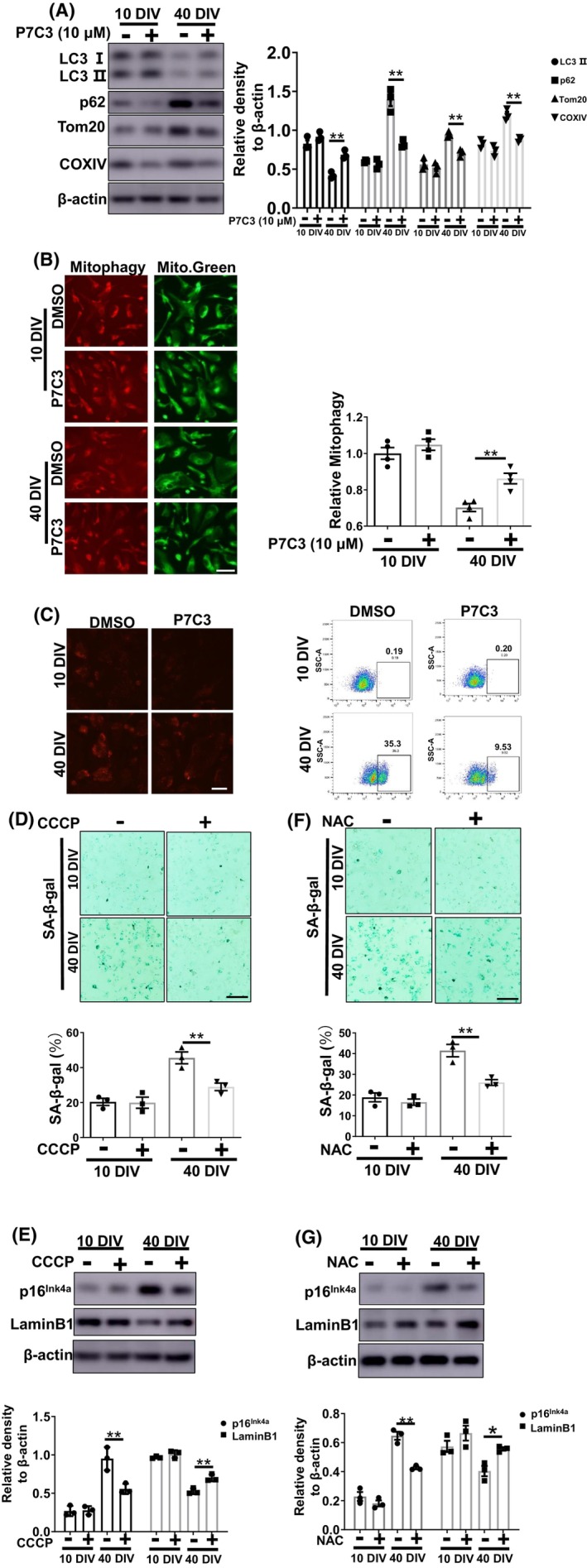
Enhanced mitophagy is involved in P7C3‐mediated astrocytic senescence inhibition. (A) Astrocytes were cultured for 10 or 40 days in vitro (DIV) and then treated with P7C3 (10 μM) for 5 days. The protein levels of LC3, p62, Tom20, COXIV, and β‐actin were measured using immunoblot analysis. The intensity quantification of LC3 II, p62, Tom20, COXIV relative to β‐actin is shown in the right panel. The values are presented as the mean ± SEM. **p < 0.01 one‐way ANOVA followed by Dunnett's multiple‐comparisons test. (B) Astrocytes were treated as described in (A), the mitophagy level was measured using Mitophagy Detection Kit. The relative mitophagy level was quantificated in the right panel. The values are presented as the mean ± SEM. **p < 0.01 one‐way ANOVA followed by Dunnett's multiple‐comparisons test. Scale bar, 10 μm. (C) Astrocytes were treated as described in (A), mitochondrial reactive oxygen species (MitoROS) was detected using MitoSox Red. The fluorescence intensity of MitoROS was also elevated by flow cytometer. Scale bar, 10 μm. (D, E) Astrocytes were cultured for 10 days or 40 days in vitro (DIV) and then treated with CCCP for 5 days. The SA‐β‐gal was stained and the percentage of SA‐β‐gal^+^ cells was shown in the panel below (D). Scale bar, 50 μm. The protein levels of p16^Ink4a^, LaminB1, and β‐actin were detected by immunoblotting and the intensity quantification of p16^Ink4a^ and LaminB1 relative to β‐actin is shown in the panel below (E). (F, G) Astrocytes were cultured for 10 or 40 days in vitro (DIV) and then treated with NAC for 5 days. The SA‐β‐gal was stained and the percentage of SA‐β‐gal^+^ cells was shown in the panel below (F). Scale bar, 50 μm. The protein levels of p16^Ink4a^, LaminB1, and β‐actin were detected by immunoblotting and the intensity quantification of p16^Ink4a^ and LaminB1 relative to β‐actin is shown in the panel below (G). The values are presented as the mean ± SEM. **p < 0.01 one‐way ANOVA followed by Dunnett's multiple‐comparisons test. *p < 0.05, one‐way ANOVA followed by Dunnett's multiple‐comparisons test.

Next, we explored the mechanism by which P7C3 inhibited astrocyte senescence. Accumulating evidence indicates that mitochondrial dysfunction plays an essential role in cellular senescence.^29^, ^30^ In our previous study, P7C3 protected mitochondrial function.^24^ It has been reported that mitophagy dysfunction and excess mitochondrial ROS accelerate cellular senescence.^31^, ^32^ Therefore, we evaluated mitophagy‐related proteins, including Tom20 and COXIV. We discovered significantly higher Tom20 and COXIV protein levels in senescent astrocytes compared to non‐senescent cells, as well as decreased microtubule‐associated protein light chain 3 (LC3)‐II and higher p62 expression in senescent astrocytes (Figure 4A). Interestingly, P7C3 reversed these changes and promoted mitophagy in senescent astrocytes (Figure 4A,B; Figure S3). Moreover, we used mitoSOX to detect mitochondrial ROS levels in senescent astrocytes. P7C3 treatment inhibited the enhanced mitochondrial ROS production (Figure 4C). To further determine the role of mitophagy promotion and mitochondrial ROS elimination in inhibiting astrocytic senescence, CCCP (a mitophagy inducer) and NAC (an oxygen‐free radical scavenger) were utilized. Both CCCP (Figure 4D,E) and NAC (Figure 4F,G) alleviated astrocytic senescence, as shown by decreased levels of β‐galactosidase and p16^Ink4a^ and increased LaminB1 levels in senescent astrocytes. These data suggested that P7C3 promoted mitophagy and inhibited mitochondrial ROS over‐production to result in astrocytic senescence mitigation.

### 3.5 P7C3 promoted SIRT3 expression to enhance mitophagy and decrease mitoROS

**FIGURE 5:**
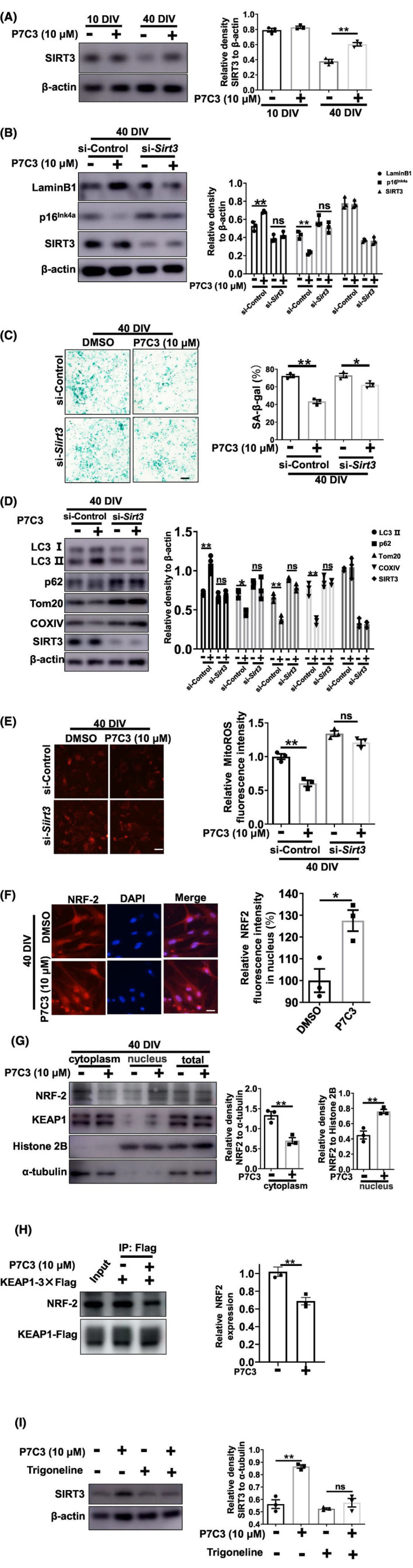
P7C3 Promoted Sirt3 expression to enhance mitophagy and decrease mitochondrial ROS. (A) Astrocytes were cultured in vitro for 10 or 40 days and then treated with P7C3 (10 μM) for 5 days. The protein levels of SIRT3 and β‐actin were measured using immunoblot analysis. The intensity quantification of SIRT3 relative to β‐actin is shown in the right panel. The values are presented as the mean ± SEM. **p < 0.01 one‐way ANOVA followed by Dunnett's multiple‐comparisons test. (B) Astrocytes were cultured in vitro for 40 days, the were transfected with control siRNA or *Sirt3* siRNA for 48 h, and then exposed to P7C3 for 5 days. The protein levels of LaminB1, p16^Ink4a^, SIRT3, and β‐actin were measured using immunoblot analysis. The intensity quantification of LaminB1, p16^Ink4a^, SIRT3 relative to β‐actin is shown in the right panel. The values are presented as the mean ± SEM. **p < 0.01, ns, no significance, one‐way ANOVA followed by Tukey's multiple‐comparisons test. (C) Astrocytes were treated as described in (B), the SA‐β‐gal was stained and the percentage of SA‐β‐gal^+^ cells was shown in the right panel. *p < 0.05, **p < 0.01 one‐way ANOVA followed by Tukey's multiple‐comparisons test. Scale bar, 50 μm. (D) Astrocytes were treated as described in (B), the protein levels of LC3, p62, Tom20, COXIV, SIRT3 and β‐actin were measured using immunoblot analysis. The intensity quantification of LC3 II, p62, Tom20, COXIV, SIRT3 relative to β‐actin is shown in the right panel. The values are presented as the mean ± SEM. *p < 0.05, **p < 0.01, ns, no significance, one‐way ANOVA followed by Tukey's multiple‐comparisons test. (E) Astrocytes were treated as described in (B), mitochondrial reactive oxygen species (MitoROS) was detected using MitoSox Red. The relative quantification of MitoROS is shown in the right panel. **p < 0.01, ns, no significance, one‐way ANOVA followed by Dunnett's multiple‐comparisons test. Scale bar, 10 μm. (F) Astrocytes were treated as described in (A). Immunofluorescence staining was performed with anti‐NRF2 and DAPI, relative NRF2 fluorescence intensity in nuclues is shown in the right panel. *p < 0.05, Student's *t*‐test. Scale bar, 5 μm. (G) Astrocytes were treated as described in (A). Then, the cytoplasmic and nuclear fractions were separated using subcellular fractionation methods. The NRF2 protein levels in cytoplasm and nucleus were detected using immunoblot analysis. The quantitative analyses of the relative density of NRF2 to loading controls in cytoplasm (α‐Tubulin) or in the nucleus (Histone 2B) were shown in the right panels. The values were presented as the means ± SEM from three independent experiments. **p < 0.01, Student's *t*‐test. (H) Astrocytes were treated as described in (A), Immunoprecipitation (IP) shows the protein levels of NRF2 and KEAP1‐Flag in astrocytes with indicated treatment. An anti‐Flag antibody was used for IP. 10% total protein used as input. **p < 0.01, Student's *t*‐test. (I) Astrocytes were cultured in vitro for 40 days, then were treated with or without Trigoneline for 24 h, followed by the treatment of P7C3 for 5 days. The protein levels of SIRT3 and β‐actin were measured using immunoblot analysis. The intensity quantification of SIRT3 relative to β‐actin is shown in the right panel. The values are presented as the mean ± SEM. **p < 0.01, ns, no significance, Student's *t*‐test.

We assessed the mechanism by which P7C3 induced mitophagy and inhibited mitoROS. SIRT3, a mitochondria‐localized sirtuin, possesses a robust ability to inhibit cellular senescence.^33^, ^34^ SIRT3 was substantially downregulated in senescent astrocytes (Figure 5A). We tested whether P7C3 could inhibit astrocytic senescence by affecting SIRT3 expression. Notably, the reduced SIRT3 expression in senescent astrocytes was largely reversed with P7C3 treatment (Figure 5A). To confirm the central role of SIRT3 in P7C3‐mediated inhibition of astrocytic senescence, we knocked down *Sirt3* in senescent astrocytes. The inhibition of p16^Ink4a^ and β‐galactosidase expression by P7C3 and the promotion of LaminB1 expression were largely abolished in *Sirt3*‐knockdown astrocytes (Figure 5B,C), indicating that SIRT3 is involved in P7C3‐modulated anti‐senescence in astrocytes.

P7C3 inhibition of astrocytic senescence directly depended on the promotion of mitophagy and inhibition of mitoROS production. Therefore, we determined whether the regulation of mitophagy and mitoROS by P7C3 was effective in *Sirt3*‐deficient astrocytes. Notably, P7C3‐mediated downregulation of p62, Tom20, COXIV, and mitoROS and upregulation of LC3‐II expression were almost completely eliminated in *Sirt3*‐deficient astrocytes (Figure 5D,E). These observations suggested that the regulation of mitophagy and mitoROS production relied on the presence of SIRT3.

Next, we assessed the mechanism by which P7C3 facilitated SIRT3 expression. It has been reported that nuclear respiratory factor 2 (NRF2) induces SIRT3 expression by binding to directly the *Sirt3* promoter.^35^, ^36^ NRF2 needs to enter the nucleus to perform its function. We determined whether P7C3 promoted nuclear translocation of NRF2, which would induce SIRT3 expression. In our nucleo‐cytoplasmic separation experiments and immunofluorescence experiments, we indeed found that P7C3 accelerated the nuclear translocation of NFR2 (Figure 5F,G). As a downstream of NRF2, the expression of HO‐1 also increased significantly after P7C3 treatment (Figure S2C). The E3 ligase adaptor KEAP1 acts as a primary negative regulator for NRF2. Therefore, we conducted experiments to investigate if P7C3 could interfere with the formation of the NRF2‐KEAP1 protein complex. In the KEAP1 immunoprecipitation (IP) assay, treatment with 10 μM P7C3 partially decreased the interaction between NRF2 and KEAP1 (Figure 5H). Interestingly, trigonelline, a novel NFR2 inhibitor, robustly reversed P7C3‐induced SIRT3 upregulation (Figure 5I). Thus, these data indicated that the NRF2/SIRT3 axis is closely associated with P7C3‐mediated mitophagy in senescent astrocytes.

## 4 DISCUSSION

P7C3 exhibits powerful neuroprotective effects in several neurodegenerative diseases, including PD and AD.^22^, ^23^, ^25^ It was discovered that the neuroprotective effects of P7C3 primarily depended on the activation of NAMPT,^37^ the rate‐limiting enzyme for NAD synthesis. Subsequently, P7C3 has emerged as a NAMPT activator in various disease studies, including peripheral neuropathy^38^ and diabetes.^39^ Recently, several studies have found that P7C3 significantly inhibits neuroimmune system overactivation by regulating glial activation.^40^, ^41^ P7C3 also limits the progression of glioma by suppressing aerobic glycolysis.^42^ In our previous study, we determined that P7C3 inhibited LPS‐induced microglial activation, protecting DA neurons from death.^26^ However, the regulatory role of P7C3 on glial cells remained to be explored. In this study, we discovered a novel regulatory effect of P7C3 on glial cells, namely, P7C3 inhibited astrocytic senescence and alleviated PD progression.

Glial senescence has been identified as a critical component of age‐related neurodegenerative diseases.^5^ Astrocytes, the most abundant glial cells in the brain, have a crucial role in maintaining homeostasis within the CNS. They provide nutrients to neighboring neurons, regulate synaptic plasticity, control neurotransmitter release, and preserve the integrity of the blood–brain barrier.^43^ Research has indicated that as the body ages physiologically, the expression of specific astrocytic genes in certain brain regions is altered significantly, particularly in the hippocampus and substantia nigra.^44^ Senescent astrocytes exhibit an SASP and impaired functions in lysosomes, mitochondria, neurotransmitter transport, lipid metabolism, and neurotrophic factor secretion. Impairment of these characteristics can potentially harm DA neurons.^45^ Paraquat, a neurotoxin that induces PD, promotes astrocytic senescence. In a paraquat‐induced PD animal model, targeting senescent astrocytes through clearance or inhibition improved movement symptoms associated with the disease.^11^ These studies reveal a robust correlation between astrocytic senescence and the progression of PD.

Recent studies have reported that mitochondrial dysfunction is critical for cellular senescence.^46^, ^47^ During aging, astrocytes experience impaired mitophagy and excessive ROS release from mitochondria.^48^ Therefore, reducing mitochondrial ROS production or promoting the removal of damaged mitochondria could significantly decrease the expression of aging traits.^27^ This observation highlights the critical role of maintaining mitochondrial homeostasis in preventing senescence. Our previous study showed that P7C3 inhibits mitochondria‐mediated apoptosis by inhibiting GSK3β activation.^24^ Thus, there may be additional connections between P7C3 and mitochondrial function.

**FIGURE 6:**
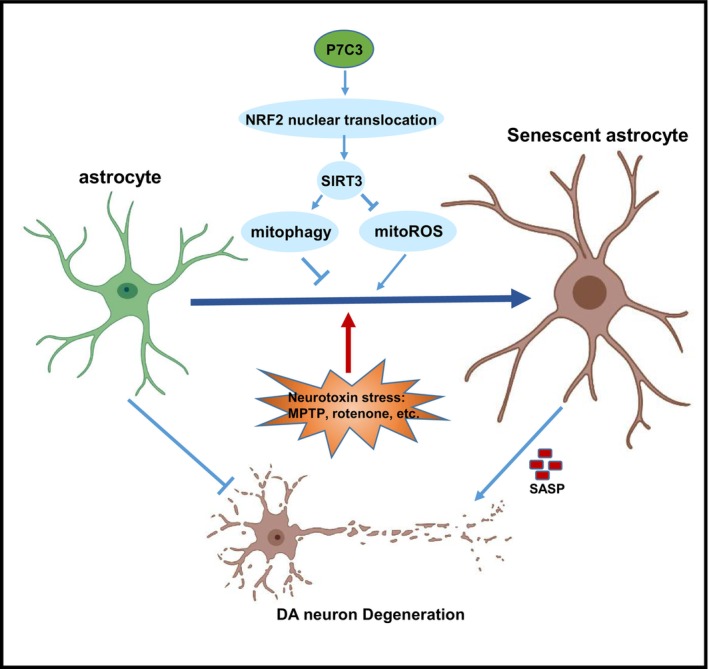
A schematic diagram illustrates the role of mitophagy promotion and mitoROS inhibition in the suppression of astrocytic senescence by P7C3. When astrocytes are exposed to neurotoxins (MPTP or rotenone), they undergo senescence and release excessive SASP factors such as IL‐6, IL‐1β, CXCL10, and MMP9. These SASP factors can harm nearby dopaminergic neurons. However, with the P7C3 treatment, senescent astrocytes could promote NRF2 nuclear translocation, leading to an increase in SIRT3 expression. This, in turn, further promotes mitophagy and inhibits mitoROS production, ultimately protecting dopaminergic neurons from damage.

This study revealed the molecular mechanism responsible for P7C3's suppressive effects on astrocytic senescence (Figure 6). NRF2/SIRT3‐mediated mitophagy and inhibition of mitoROS production were closely associated with P7C3‐mediated astrocytic senescence inhibition. SIRT3, a sirtuin located in mitochondria, is crucial in maintaining mitochondrial homeostasis. SIRT3 suppresses the production of mitoROS and facilitates Parkin/PINK1‐mediated mitophagy.^49^ The effects of P7C3 on SIRT3 may depend on the P7C3‐mediated NAMPT‐NAD^+^ regulatory axis, as the acetylation activity of SIRT3 relies on the supply of NAD^+^. However, our findings in astrocytes suggested that P7C3 could promote NRF2 nuclear translocation directly, which, in turn, facilitated SIRT3 transcription, leading to mitophagy and mitoROS clearance. While the nuclear entry of NRF2 is dependent on the degradation of KEAP1, our results indicated that P7C3 does not influence KEAP1 expression (Figure S2D). Additional research is needed to determine whether and how P7C3 can directly or indirectly promote the dissociation of NRF2 from KEAP1.

The protective effect of P7C3 on DA neurons is not solely reliant on its inhibitory effect on astrocytic senescence. Our previous study revealed that the inhibition of microglial activation by P7C3 also significantly contributed to the protection of DA neurons.^26^ Additionally, P7C3 has the ability to directly maintain mitochondrial function to protect DA neurons.^24^ Our present study enhances the understanding of P7C3's protective effects on DA neurons by exploring the role of astrocytic senescence. Building upon our previous research, we suggest that P7C3's protection of DA neurons may involve both direct prevention of mitochondrial damage and indirect suppression of microglia activation and astrocytic senescence.

Our current study has some limitations. Previously, we utilized pretreatment with P7C3 in experiments involving PD mouse model,^24^ and this method was also employed in this study to investigate the impact of P7C3 on astrocytic senescence. Typically, drug treatment is administered after the onset of PD. Additionally, studies have shown that in the 6‐OHDA‐induced rat model of PD, rats were initially exposed to 6‐OHDA followed by treatment with P7C3, leading to a significant neuroprotective effect.^25^ Moving forward, we aim to assess whether P7C3 post‐conditioning also exhibits substantial neuroprotective effects in the MPTP‐induced mouse model of PD.

In summary, this study demonstrated that P7C3 significantly suppressed replicative astrocytic senescence and neurotoxin‐induced premature astrocytic senescence in vitro and in vivo.

## AUTHOR CONTRIBUTIONS

All authors had access to all study data and were responsible for data integrity and accuracy of data analysis. Chao Gu, Mei Wang, Yajing Chen, and Guanghui Wang contributed to the study conception and design. Zengyan Zhu, Yinghui Yan, Hongyang Sun, Xiaohuan Du, Fang Li, Shuwei Yuan, Wenjing Wang contributed to material preparation, model establishment, data collection, and analysis. Chao Gu, Mei Wang, Yajing Chen, and Guanghui Wang contributed to discussion. The first draft of the manuscript was written by Chao Gu and Yajing Chen, and all the authors commented on previous versions of the manuscript.

## FUNDING INFORMATION

National Natural Science Foundation of China (Grant Number: 32200775); Jiangsu Innovative and Enterpreneurial Talent Programme (Grant Number: (2021) 31576); Suzhou Medical Health Science and Technology Innovation Project (Grant Number: SKJY2021103); The Suzhou Science and Technology Project (Grant Number: SKY2022169). Suzhou Health Talent Plan Talent Research Project (Grant Number: GSWS2023102).

## CONFLICT OF INTEREST STATEMENT

The authors declare that they have no competing interests.

## Supporting information


Data S1.

## Data Availability

The data that support the findings of this study are available from the corresponding author upon reasonable request.
