# Supplementary material for: P7C3 suppresses astrocytic senescence to protect dopaminergic neurons: Implication in the mouse model of Parkinson’s disease
Source: CNS Neurosci Ther. 2024 Jul 26;30(7):e14819. doi: 10.1111/cns.14819 (PMC11273101; doi:10.1111/cns.14819)
Supplement: Supplementary file 1 — Data S1. [file CNS-30-e14819-s001.docx]

**P7C3 suppresses astrocytic senescence to protect dopaminergic neurons: implication in the mouse model of Parkinson's Disease**


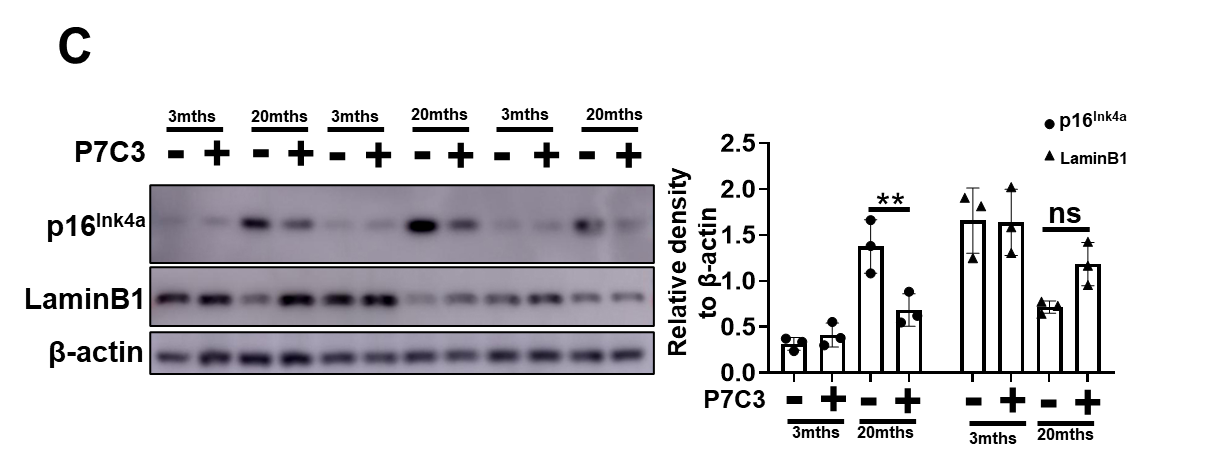

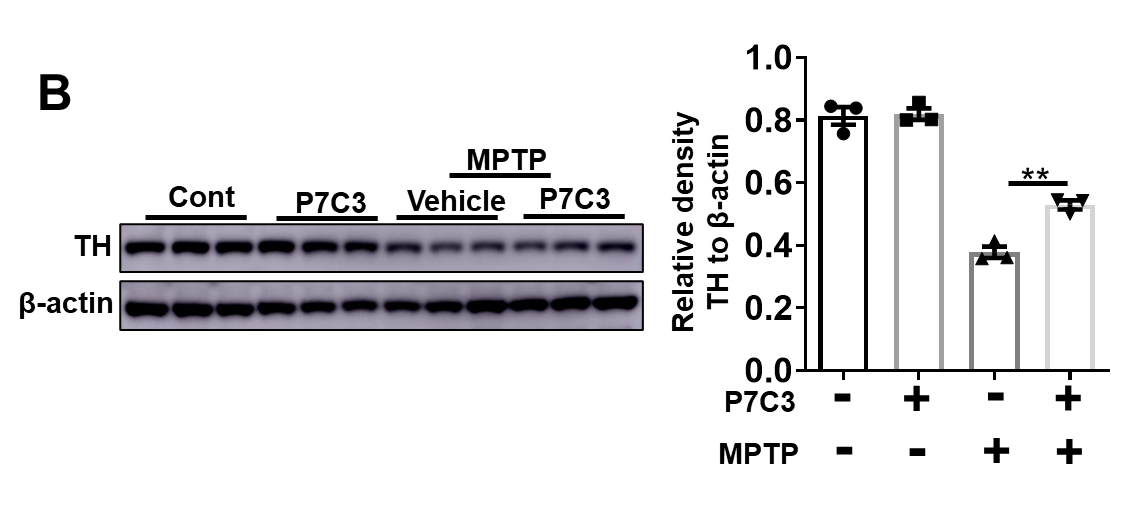

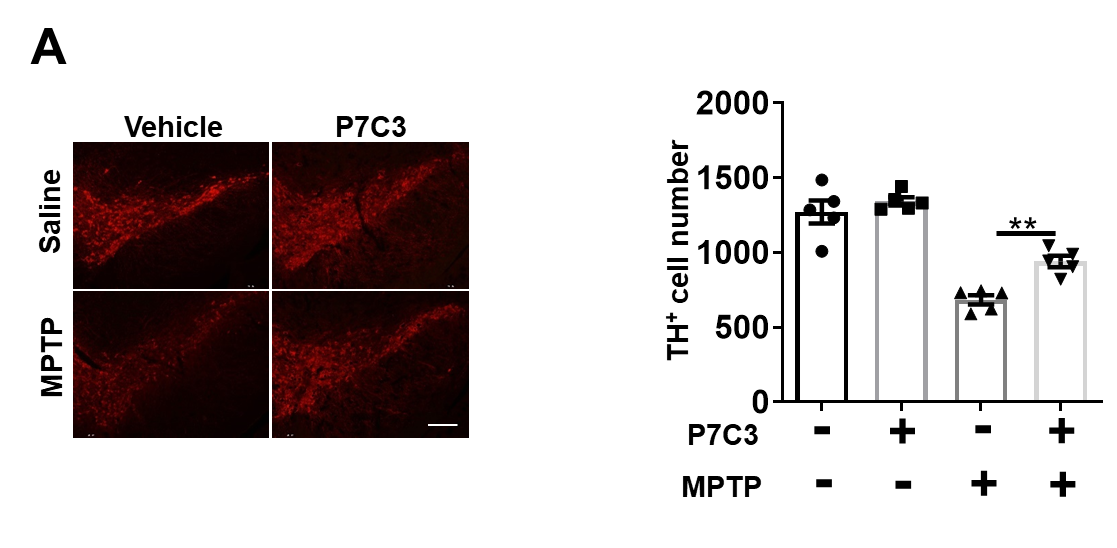
***Supplementary information***

**Supplementary Figure 1.** **(A)** Immunohistochemical staining was performed using anti-TH antibodies. The quantification of TH^+^ cell numbers is presented in the right panel. The scale bar represents 100 μm. The sample size was five per group. ***P* < 0.01 compared to the group treated with MPTP alone, determined using one-way ANOVA followed by Dunnett’s multiple-comparisons test. **(B)** Mice midbrain was isolated to collect total protein. The protein levels of TH and β-actin were detected by immunoblotting. The intensity quantification of TH relative to β-actin is shown in the right panel. ***P* < 0.01 compared to the group treated with MPTP alone, determined using one-way ANOVA followed by Dunnett’s multiple-comparisons test. **(C)**Three months-old and twenty-months-old C57BL/6 mice were purchased from SLACCAL Lab Animal Ltd. (Shanghai, China). The mice were raised with 50-60% relative humidity, a temperature of 20-26 ℃, and a 12:12 h light/dark cycle. The animals were provided water and food *ad libitum*. For P7C3 treatment, the mice were randomly divided into four groups: (1) vehicle + 3 months-old, (2) P7C3 + 3 months-old, (3) vehicle + 20 months-old, and (4) P7C3 + 3 months-old. The vehicle was composed of 3% DMSO/10% cremophor EL/87.5% D5W (5% dextrose in water, pH 7.2). The mice in groups (2) and (4) were treated twice daily with 20 mg/kg/d P7C3 for 21 consecutive days via intraperitoneal injection. The mice were euthanized 24 hours after last treatment. Mice midbrain was isolated to collect total protein. The protein levels of p16^Ink4a^, LaminB1 and β-actin were detected by immunoblotting. The intensity quantification of p16^Ink4a^ and LaminB1 relative to β-actin is shown in the right panel. ***P* < 0.01 or ns, no significance compared to the 20 months-old mice group without P7C3 treatment, determined using one-way ANOVA followed by Dunnett’s multiple-comparisons test.


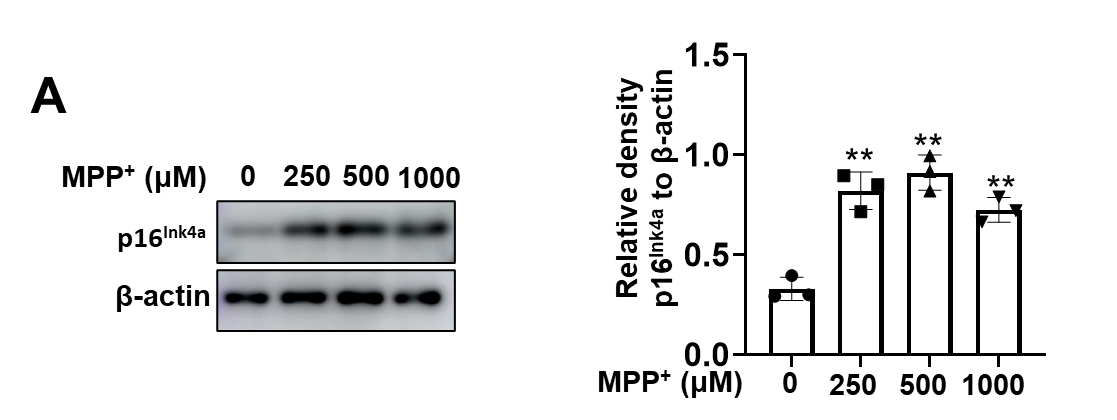


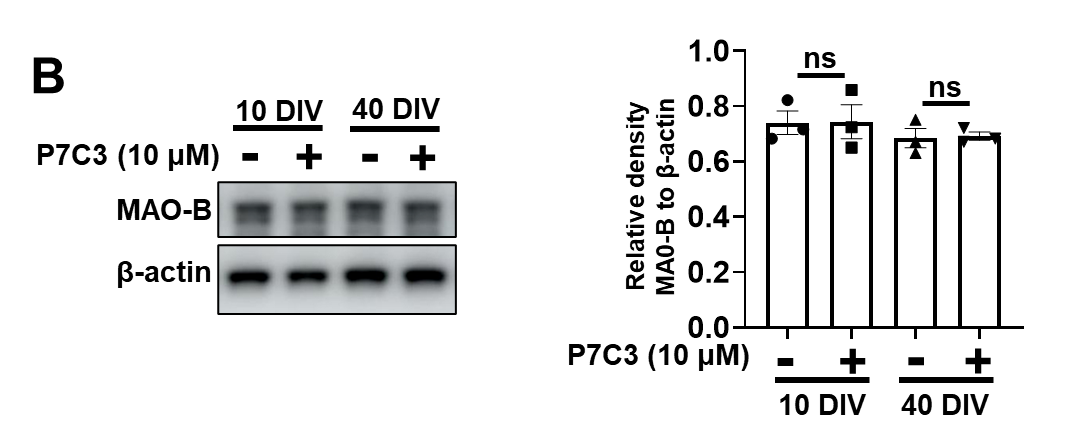


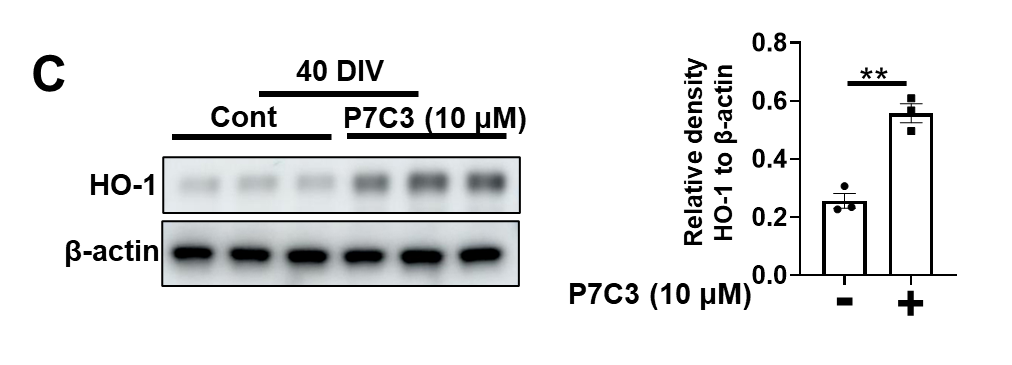


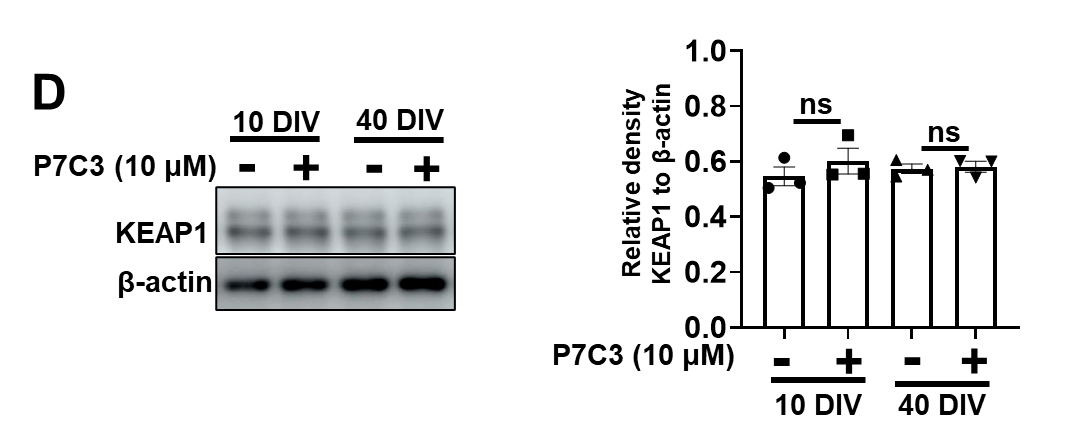


**Supplementary Figure 2.** **(A)** Astrocytes were exposed to MPP^+^ (250, 500, 1000μM) for 24 h. The protein levels of p16^Ink4a^ and β-actin and actin were measured using immunoblot analysis. The intensity quantification of p16^Ink4a^ relative to β-actin is shown in the right panel. The values are presented as the mean ± SEM. ***P* < 0.01, one-way ANOVA followed by Dunnett’s multiple-comparisons test. **(B)** Astrocytes were cultured *in vitro* for 10 or 40 days and then treated with P7C3 (10 μM) for 5 days. The protein levels of MAO-B and β-actin were measured using immunoblot analysis. The intensity quantification of MAO-B relative to β-actin is shown in the right panel. The values are presented as the mean ± SEM. ns, no significance one-way ANOVA followed by Tukey’s multiple-comparisons test. **(C)** Astrocytes were cultured *in vitro* for 40 days and then treated with P7C3 (10 μM) for 5 days. The protein levels of HO-1 and β-actin were measured using immunoblot analysis. The intensity quantification of HO-1 relative to β-actin is shown in the right panel. The values are presented as the mean ± SEM. ***P* < 0.01 by Student’s t test. **(D)** Astrocytes were cultured in vitro for 10 or 40 days and then treated with P7C3 (10 μM) for 5 days. The protein levels of KEAP1 and β-actin were measured using immunoblot analysis. The intensity quantification of KEAP1 relative to β-actin is shown in the right panel. The values are presented as the mean ± SEM. ns, no significance one-way ANOVA followed by Tukey’s multiple-comparisons test.

**Supplementary Figure 3.**
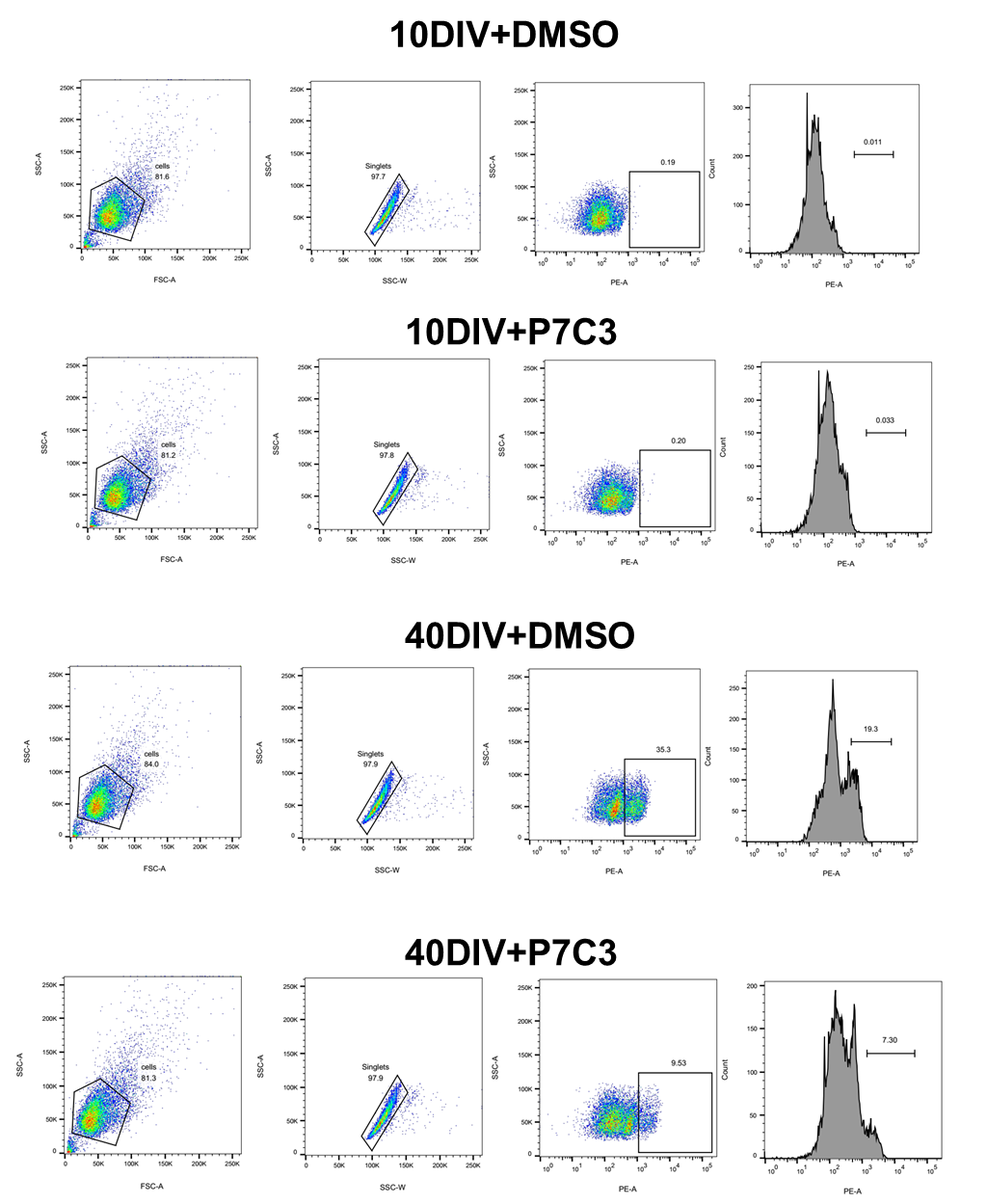
 Detailed gating information on flow cytometry in Figure 4C.
